# Vaccine hesitancy in Brazil: post-pandemic challenges and the use of artificial intelligence

**DOI:** 10.11606/s1518-8787.2026060007418

**Published:** 2026-07-17

**Authors:** Ana Paula Sayuri Sato

**Affiliations:** I Universidade de São Paulo. Faculdade de Saúde Pública. Departamento de Epidemiologia. São Paulo, SP, Brasil

**Keywords:** Vaccines, Immunization Programs, Vaccination Refusal, Disinformation, Generative Artificial Intelligence

## Abstract

Vaccine hesitancy is a global public health challenge, and its characteristics change according to the social and epidemiological context. This commentary seeks to stimulate debate on the dynamic nature of the definition and determinants of vaccine hesitancy, especially in the wake of the Covid-19 pandemic and the population’s increased access to new forms of information-seeking, such as artificial intelligence. Scientific production on childhood vaccine hesitancy in Brazil before 2016 was focused on high-income families. After 2016, there was an increase in production on vaccine hesitancy, considering the World Health Organization’s 2014 determinants model. After the start of the pandemic, there was a significant increase in studies on disinformation and the politicization of the vaccine. Currently, the use of generative artificial intelligence, which is increasingly common in the search for information on vaccines, is being considered.

## INTRODUCTION

Vaccine hesitancy was considered one of the ten most relevant global health threats in 2019 by the World Health Organization (WHO). It is a complex phenomenon, with multiple determinants and which changes according to the social and epidemiological context, varying in relation to time, place, and types of vaccines^1^.

Brazil’s National Immunization Program is more than 50 years old and offers around 50 immunobiologicals free of charge and universally, with 32 vaccines that cover people from birth at all stages of life. The drop in vaccination coverage in Brazil was evident after 2016, with extensive scientific literature produced to portray a situational diagnosis, populations most affected and determinants. With the onset of the Covid-19 pandemic, several studies have pointed to a worsening drop in coverage, indicating, in addition to vaccine hesitancy, worsening access to vaccines in the country^2,3^. Current data from the National Immunization Program show an improvement in vaccination coverage in 2024, but outside the range of targets for most vaccines.

The growth of vaccine hesitancy in the Brazilian population is pointed out as one of the determinants of the drop in vaccination coverage. Data from the Vaccine Confidence Project for Brazil from 2015 to 2019 highlights the drop in the proportion of people who strongly agree on the importance (average of 88.3%), safety (average of 66.6%), and effectiveness of vaccines (average of 55.9%)^4^. The 2007 National Vaccination Coverage Survey showed that high-income families had lower vaccination coverage^5^. In 2020, the Survey, which covered state capitals, the Federal District, and 12 other municipalities, showed a high frequency of vaccine hesitancy in the country, being higher in families from lower social strata and lower maternal schooling. However, access barriers (transportation, time, lack of knowledge, and loss of the vaccination booklet) and programmatic difficulties (queues and lack of recommendations) were the most relevant factors for lower vaccination coverage^6^. After the start of the Covid-19 pandemic, the increase in disinformation through social media and the use of artificial intelligence by the population may have affected the profile of the hesitant population.

The aim of this commentary is to stimulate debate on the dynamic nature of the definition and determinants of vaccine hesitancy, especially after the Covid-19 pandemic and with the population’s increased access to new forms of information-seeking, such as artificial intelligence.

### The Dynamic Concept of Vaccine Hesitancy and Its Determinants

The term “vaccine hesitancy” was established in 2012–2014, when the WHO set up an expert group, which defined it as the “delay in acceptance or refusal of vaccination despite availability of vaccination services”. On that occasion, the three Cs model (confidence, complacency, and convenience) and the matrix of contextual, individual and vaccine/vaccination-specific determinants were presented^1^.

In 2018, Betsch et al.^7^ proposed a five-C model on the psychological antecedents of vaccination to support strategies for monitoring and intervening in vaccine hesitancy around the world. The five Cs correspond to confidence (vaccine efficacy, and safety), complacency (lower perceived disease risk), constraints (structural and psychological barriers), calculation (engagement in the search for information and cost-benefit analysis), and collective responsibility (willingness to protect others). It should be noted that information-seeking engagement can lead to greater or lesser vaccine hesitancy depending on the sources used, due to the wide availability of anti-vaccine content and disinformation on social media and the internet. The term “convenience” has been replaced by “constraints”, as the former shifts responsibility to the individual, such as low prioritization of vaccination, without considering determinants of the social process. It also works with the issue of access as a separate construct.

The term “hesitancy” was also debated, due to its behavioral definition (delay in acceptance or refusal vaccines), since this phenomenon can be a psychological state of indecision. Understood as a behavior, vaccine hesitancy has been indirectly assessed in many studies as non-vaccination even when the reason was related to access difficulties, health system failures or even outright refusal of any vaccine^7,8^.

Many studies focus on identifying groups/areas or characteristics associated with lower vaccination coverage; however, it is necessary to understand the potentially modifiable determinants to actually increase vaccination. In 2018, the WHO created a working group to study the Behavioral and Social Drivers (BeSD) of vaccination, initially focused on childhood vaccines and, as a result of the pandemic, expanded to Covid-19 vaccines among adults and health professionals^9^. The work of Brewer et al.^10^ was used as a reference, proposing the Increasing Vaccination Model, based on behavioral science, to help tackle low vaccination coverage by identifying effective interventions.

Vaccine uptake is the result of a complex network of connections between different actors, resources and behaviors. In the first domain, people’s thoughts/feelings about vaccination are considered, which includes the perceived disease risk and confidence in vaccine safety and efficacy. It is worth noting that this domain is strongly influenced by previous experiences with vaccination, whether their own or those of others, such as adverse events, and by the quality of the information accessed. This domain motivates people to get vaccinated or not. Low motivation to get vaccinated can be referred to as “vaccine hesitancy”^10^.

Another domain that affects motivation to get vaccinated is the social process that involves connections between people, especially the role of family members and community or religious leaders, establishing social norms (what people are expected to do) and social preferences (getting vaccinated to protect others or not getting vaccinated because others have already been vaccinated)^10^.

A third domain is direct behavior change, which assumes that motivation is already present and that it is possible to increase vaccination without necessarily changing what people think/feel or the social context in which they live. This domain can be worked on by building intentions that are favorable to vaccination, such as keeping vaccination present in people’s daily lives through reminders and media stimuli and reducing barriers to access, as well as through incentives or requirements that promote vaccination^10^.

It was observed that interventions that try to modify the thinking/feeling domain, although correlated with receiving the vaccine, show less consistent effects. Interventions in the social process domain are promising but still require more evidence. Interventions in the area of direct behavioral change have more significant effects on increasing vaccination. Finally, recommendation by health professionals stands out as the most powerful intervention^10^.

In 2022, the WHO published a position paper on the results of the working group, presenting an adaptation of the Increasing Vaccination Model (Figure). Vaccine hesitancy was defined as “a motivational state of being conflicted about, or opposed to, getting vaccinated; this includes intentions and willingness”. It separates the motivation/hesitancy from the resulting behavior (receiving the vaccine), so that they should be measured and understood independently. This definition replaces the previous one from 2014^1,9^.

**Figure:**
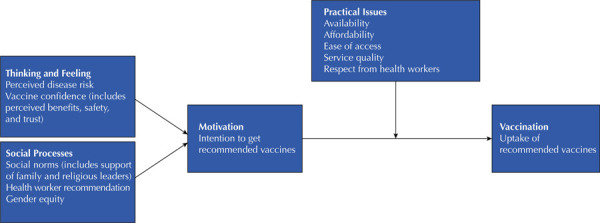
World Health Organization framework on the behavioral and social determinants of vaccination, 2022.

Source: Adapted from WHO^9^ (2022).

### Different Stages of Childhood Vaccine Hesitancy in Brazil

In Brazil, before the WHO defined vaccine hesitancy in 2014 and the drop in vaccination coverage in 2016, i.e. a time of high vaccination coverage, scientific production on vaccine refusal or delay was more significant in qualitative studies of middle- and high-income families and showed the determination of the social process on the decision to (not) vaccinate^11,12^.

With the need to understand the drop in vaccination coverage, since 2016, studies on vaccine hesitancy in Brazil have grown in number and started to consider the three Cs models and the WHO matrix of determinants^13,14^. The study by Brown et al.^13^ showed that the most common reasons for hesitancy were issues related to confidence in the vaccine. Silveira et al.^15^ demonstrated the emergence of vaccine hesitancy in the birth cohorts of Pelotas (RS), based on the reversal of the pattern of the social gradient from lower vaccination coverage among the poorest families in 1982 to the richest in 2015. In Campo Grande (MT), Nascimento et al.^14^ found that 39% of parents were hesitant, mainly due to a lack of confidence in the vaccine. Garcia et al.^16^ used the WHO 10-item Likert scale instrument, the Vaccine Hesitancy Scale^1^, in Araraquara (SP). They distinguished between two components – vaccine confidence and the perceived adverse events risk – and highlighted the role of health professionals in dealing with vaccine hesitancy, guiding vaccination through a relationship of trust^16^.

The 2020 Vaccination Coverage Survey showed that access barriers and programmatic difficulties supplanted vaccine hesitancy as the reason for lower adherence to vaccination. The two most obvious access reasons that explained the 62% reduction in full vaccination coverage were the lack of money for transportation and the loss of the vaccination booklet^6,17^.

Qualitative studies are also noteworthy, such as Matos et al.^18^, who highlighted the role of the social context, health professionals and access to information as being fundamental to the decision-making process regarding childhood vaccination. The perception of risk and confidence in the vaccine (composition, effectiveness, and adverse events), in the PNI (vaccination schedule), and in the pharmaceutical industry, as well as families’ lifestyles and worldview, were key elements in vaccine hesitancy. Cunegundes et al.^19^ worked with highly educated parents in São Paulo (SP) who dismissed the anti-vaccine label. They discussed (non-)vaccination as part of a set of choices that characterize a broader social identity and four themes emerged from the narratives: natural lifestyle; social pressure in the context of vaccination; pandemic and vaccination against Covid-19; and distrust of vaccines, the pharmaceutical industry, health professionals and institutions.

Several studies on Covid-19 vaccine hesitancy have indicated a prevalence of hesitancy of around 20% in the general population^20,21^ and for specific groups such as children^22^, pregnant women^23^, the elderly^24^, and specific clinical conditions^25^. In these studies, in order to analyze the association, in addition to variables related to vaccine confidence and perceived disease risk, socioeconomic and demographic variables, the characteristics of the vaccines (type of platform, laboratories and country of origin), comorbidities, religion, political orientation and ways of seeking information, with an emphasis on online media^20,21,23,26^ were analyzed more forcefully.

The recommendation of vaccination by health professionals is widely recognized as one of the most effective actions for increasing adherence to the vaccine. However, vaccine hesitancy among health professionals has also been the subject of several studies and reveals a worrying scenario, both for vaccination itself^27,28^, and for the recommendation of vaccination for the population^29,30^.

After the pandemic, studies on vaccine hesitancy and political and religious orientation stand out^26,31,32^. A study by Pereira et al.^26^ highlighted that religion plays an important role in affecting political preferences and the perception of vaccine safety. Matos et al.^33^ introduced the term “vaccine politicization”, defining it as “the influence of political interests and ideologies over technical or scientific evaluations on vaccines policies, aiming to define or modifying public opinions in favor of a political interest”. From the perspective of children’s caregivers, the vaccine politicization became evident with the Covid-19 pandemic and should be treated as a contemporary challenge in the Brazilian context and considered in the formulation of immunization policies^33^.

The Covid-19 pandemic has affected the credibility of scientific institutions and the traditional media, leading to the rise of conspiratorial beliefs and authoritarian narratives. Scientific denialism has thus compromised adherence to vaccination^33^.

Disinformation about vaccines, in the face of a hyperconnected population, has become even greater with the pandemic. In English, the literature differentiates the concepts of “misinformation”, as incorrect information, but without intent, from “disinformation”, as incorrect information with deliberate intent to mislead/cause harm^34^. Galhardi et al.^34^ found that the platforms Instagram, Facebook, Twitter, and WhatsApp were the main means of sharing disinformation about Covid-19. Likewise, they detected a large circulation of disinformation about vaccines related to political polarization. A study on reasons for refusing the Covid-19 vaccine, based on an analysis of posts on Twitter, identified five thematic categories: individuality, fear of adverse events, political ideologies/aversion to government recommendations, skepticism about the vaccine’s efficacy and refusal of unnatural products or interventions. It is suggested that social media can perpetuate hesitant attitudes and intensify ideological isolation^35^. Thus, disinformation about vaccines should also be analyzed from a politicized perspective and scientific denialism. In addition, the political economy of disinformation depends on a monetization infrastructure, through hybrid fundraising strategies (donations, product sales, advertising, and monthly membership fees), to sustain itself financially and obtain economic and political incentives, which contributes to the persistence of anti-vaccine narratives^36^.

### New Challenges and Potential with the Introduction of Artificial Intelligence in the Search for Vaccine Information

As mentioned throughout the text, over the last decade and especially after the Covid-19 pandemic, the characteristics of vaccine hesitancy in Brazil have changed substantially, influenced by various factors, one of which is the way people search for and access information.

Artificial intelligence makes it possible to identify misinformation and discern patterns and feelings that influence vaccine uptake. It can be used to build innovative communication strategies to combat disinformation about vaccines, considering more vulnerable groups, those with difficulty accessing digital media, and adaptations according to psychological traits^37^.

Social media have become important sources of information and misinformation about vaccines. A sentiment analysis study based on a natural language model showed the predictive capacity of tweets on a large scale and in real time on Covid-19 vaccine coverage, demonstrating the tool’s potential for developing strategies to identify vaccine hesitancy hotspots^38^.

Salas et al.^39^analyzed ChatGPT responses to the 50 most prevalent false messages, contraindications and myths about vaccines circulating on the internet in 2023 and concluded that, although the tool cannot replace an expert, it has potential to be explored in vaccination guidance, if it uses data aligned with scientific evidence.

Randomized controlled trials have shown promising chatbot strategies to reduce vaccine hesitancy, increase vaccine confidence and correct misinformation about vaccination in various regions of the world^40^. Brown et al.^40^ showed that a Whatsapp chatbot with two-way interactive messages, with behavioral functionalities, increased Covid-19 vaccination by more than three times compared to the non-intervention group and almost doubled vaccination compared to the one-way vaccination reminder strategy. A study by Hou et al.^41^ showed the effectiveness of a chatbot aimed at human papillomavirus vaccination among elementary school girls at China. The chatbot was built using advanced linguistic technologies via GPT-4 and was based on information from health authorities. The results indicated that the chatbot increased vaccination among students and improved vaccine literacy among parents. Baudoin et al.^42^also studied the effect of chatbot intervention on adolescent students in France. They observed an improvement in attitudes towards vaccination and a reduction in mistaken beliefs. An important result was that this effect did not depend on the participants’ confidence in science, their initial attitudes towards vaccination or their academic ability. Lu et al.^43^demonstrated the potential of AI-assisted message generation to craft effective corrective messages, especially when targeting personality traits such as extroversion. However, messages addressing deep-rooted beliefs were ineffective or backfired, highlighting the need for human oversight in refining these messages.

In October 2025, the Pan American Health Organization^44^launched a guide with recommendations for prompts for generative artificial intelligence to produce public health content that is reliable and suitable for different cultures and contexts, to contribute to public health communication and decision-making. In Brazil, as a concrete experience, the Ministry of Health launched the virtual assistant on WhatsApp in December 2023, as part of the inter-ministerial Health with Science program, which focuses on valuing science and disseminating reliable information. The chatbot aims to facilitate access to correct information about vaccination (campaigns and calendars) and vaccines (characteristics, benefits and importance) and to tackle misinformation.

In addition, it is necessary to explain the challenges of using this tool in the context of unequal access to technologies, connectivity, and digital literacy. In Brazil, a study showed significant regional, demographic and social heterogeneity in access to the internet, with greater digital exclusion among the elderly, people living in poverty and residents of rural areas, and the reasons for less use included lack of interest, knowledge, availability and cost^45^. Consequently, the algorithm’s bias in using data that is not representative of the target population to train and test artificial intelligence systems stands out, perpetuating systematic discrimination and exclusion. Similarly, other biases related to opacity, privacy and data governance should be mentioned. Finally, the environmental and social impact associated with the life cycle of artificial intelligence systems, including energy resources and changes in work organization^46^should be highlighted.

Thus, with the increasingly common and easily accessible use of artificial intelligence, a new challenge has arisen in the management of vaccination adherence. On the one hand, it is possible to use this tool to increase access to correct information about vaccines and thus reduce vaccine hesitancy; on the other hand, it can generate more misinformation and inequities in vaccination by not considering the limitations of the data and algorithms used^37^.

## FINAL CONSIDERATIONS

Vaccine hesitancy is a dynamic phenomenon driven by changes in the social and epidemiological context. This commentary has recorded the evolution of the concept, as well as the way in which it has been addressed in the Brazilian scientific literature after the start of the drops in vaccination coverage in 2016 and after the start of the Covid-19 pandemic.

Artificial intelligence will have a major impact on the development of strategies to increase vaccination and a context of social inequity, and the challenges of governance and externalities of the tool must be considered. Therefore, the need to develop artificial intelligence tools for identifying, monitoring and clarifying content related to vaccination stands out, along with ways of educating the user public on how to use them properly, strengthening confidence in vaccines and reducing vaccine hesitancy.

## Data Availability

not applicable.
