# Supplementary material for: Vaccine hesitancy in Brazil: post-pandemic challenges and the use of artificial intelligence
Source: Rev Saude Publica. 2026 Jul 17;60:e29. doi: 10.11606/s1518-8787.2026060007418 (PMC13367140; doi:10.11606/s1518-8787.2026060007418)
Supplement: Translation

# Translation: A hesitação vacinal no Brasil: os desafios pós-pandemia e o uso da inteligência artificial

**Authors:** Ana Paula Sayuri Sato

**Affiliations:** I Universidade de São Paulo. Faculdade de Saúde Pública. Departamento de Epidemiologia. São Paulo, SP, Brasil

**Keywords:** Vacinas, Programas de Imunização, Recusa de Vacinação, Desinformação, Inteligência Artificial Generativa

## Abstract

A hesitação vacinal é um desafio global à saúde pública e suas características mudam conforme o contexto social e epidemiológico. Neste comentário, busca-se estimular o debate sobre o caráter dinâmico da definição e dos determinantes da hesitação vacinal, especialmente após a pandemia de covid-19 e com o aumento do acesso da população a novas formas de busca de informação, como a inteligência artificial. A produção científica sobre a hesitação vacinal infantil no Brasil antes de 2016 era focada em famílias de alta renda. Após 2016, houve aumento da produção sobre a hesitação vacinal, considerando o modelo de determinantes da Organização Mundial da Saúde de 2014. Após o início da pandemia, destaca-se o aumento expressivo de estudos sobre desinformação e politização da vacina. Atualmente, põem-se em reflexão o uso da inteligência artificial generativa, cada vez mais frequente na busca de informações sobre vacinas.

## INTRODUÇÃO

A hesitação vacinal foi considerada uma entre as dez ameaças mais relevantes de saúde global em 2019 pela Organização Mundial da Saúde (OMS). Trata-se de um fenômeno complexo, com múltiplos determinantes e que muda conforme o contexto social e epidemiológico, variando em relação ao tempo, local e tipos de vacinas^1^.

O Programa Nacional de Imunizações do Brasil possui mais de 50 anos e oferece de forma gratuita e universal cerca de 50 imunobiológicos, sendo 32 vacinas que contemplam pessoas desde o nascimento em todas as fases da vida. A queda das coberturas vacinais no Brasil foi evidente após 2016, com extensa literatura científica produzida para se retratar um diagnóstico situacional, populações mais atingidas e determinantes. Com o início da pandemia de covid-19, diversos estudos apontaram o agravamento da queda das coberturas, indicando, além da hesitação vacinal, a piora de acesso às vacinas no país^2,3^. Dados atuais do Programa Nacional de Imunizações mostram melhora das coberturas vacinais em 2024, mas fora do alcance das metas para a maioria das vacinas.

O crescimento da hesitação vacinal na população brasileira é apontado como um dos determinantes da queda das coberturas vacinais. Dados do *Vaccine Confidence Project* de 2015 a 2019, do Brasil, destacam a queda da proporção de pessoas que concordam fortemente sobre à importância (média de 88,3%), segurança (média de 66,6%) e efetividade das vacinas (média de 55,9%)^4^. O Inquérito Nacional de Coberturas Vacinais 2007 mostrou que famílias de alta renda apresentavam menores coberturas vacinais^5^. Em 2020, o Inquérito, que abrangeu as capitais de estado, distrito federal e outros 12 municípios, mostrou alta frequência de hesitação vacinal no país, sendo maior nas famílias de estrato social mais baixo e menor escolaridade materna. No entanto, destacaram-se as barreiras de acesso (transporte, tempo, desconhecimento e perda de caderneta de vacinação) e dificuldades programáticas (filas e falta de recomendação) como fatores mais relevantes para a menor cobertura vacinal^6^. Após o início da pandemia de covid-19, o aumento da desinformação, por meio de redes sociais, e do uso de inteligência artificial pela população pode ter afetado o perfil da população hesitante.

Assim, neste comentário, busca-se estimular o debate sobre o caráter dinâmico da definição e dos determinantes da hesitação vacinal, especialmente após a pandemia de covid-19 e com o aumento do acesso da população a novas formas de busca de informação, como a inteligência artificial.

### Conceito Dinâmico da Hesitação Vacinal e Seus Determinantes

O termo “hesitação vacinal” foi estabelecido entre 2012–2014, quando a OMS criou um grupo de especialistas, que a definiu como “o atraso em aceitar ou a recusa das vacinas recomendadas, apesar de sua disponibilidade nos serviços de saúde”. Nessa ocasião, foi apresentado o modelo dos três C (confiança, complacência e conveniência) e a matriz de determinantes contextuais, individuais e fatores específicos das vacinas/vacinação^1^.

Em 2018, Betsch et al.^7^ propuseram um modelo de cinco C sobre antecedentes psicológicos da vacinação para subsidiar estratégias de monitoramento e intervenção da hesitação vacinal no mundo. Os cinco C correspondem à confiança (eficácia e segurança da vacina), complacência (menor percepção de risco da doença), *constraint*s/restrições (barreiras estruturais e psicológicas), cálculo (engajamento na busca de informação e análise de custo-benefício) e c*ollective responsibility*/responsabilidade coletiva (disposição em proteger os outros). Destaca-se que o engajamento de busca de informação pode levar a maior ou menor hesitação vacinal dependendo das fontes utilizadas, devido à ampla disponibilidade de conteúdo antivacinas e de desinformação nas redes sociais e na internet. O termo “conveniência” foi substituído por “restrições”, pois o primeiro transfere a responsabilidade ao indivíduo, como baixa priorização em vacinar, não levando em conta determinantes do processo social. Além disso, trabalha com a questão de acesso como um constructo separado.

O termo “hesitação” também foi debatido, devido a sua definição comportamental (atraso em aceitar ou recusar vacinas), uma vez que esse fenômeno pode ser um estado psicológico de indecisão. Entendendo-se como comportamento, a hesitação vacinal foi avaliada indiretamente, em muitos estudos, pela não vacinação mesmo quando o motivo tenha sido em relação às dificuldades de acesso, falhas do sistema de saúde ou mesmo a recusa definitiva de qualquer vacina^7,8^.

Muitos estudos têm como foco a identificação de grupos/áreas ou características associados a menor cobertura vacinal, no entanto, é necessário compreender os determinantes potencialmente modificáveis para de fato aumentar a vacinação. Em 2018, a OMS criou um grupo de trabalho para estudar os determinantes comportamentais e sociais (*Behavioural and Social Drivers -* BeSD) da vacinação, inicialmente focado nas vacinas infantis e, em decorrência da pandemia, expandiu-se para vacinas covid-19 entre adultos e profissionais da saúde^9^. Utilizou-se como referencial o trabalho de Brewer et al.^10^, que propôs o Modelo de Aumento da Vacinação, com base na ciência do comportamento, para contribuir ao enfrentamento das baixas coberturas vacinais ao identificar intervenções efetivas.

O recebimento das vacinas resulta de uma rede complexa de conexões de diferentes atores, recursos e comportamentos. Num primeiro domínio, considera-se os pensamentos/sentimentos das pessoas sobre a vacinação, que inclui a percepção do risco da doença e a confiança na segurança e na eficácia da vacina. Vale ressaltar que esse domínio é fortemente influenciado por experiências prévias com a vacinação, sejam elas próprias ou de terceiros, como eventos adversos, e pela qualidade das informações acessadas. Tal domínio motiva as pessoas a receberem ou não a vacina. A baixa motivação em se vacinar pode ser referida como “hesitação vacinal”^10^.

Outro domínio, que afeta a motivação em se vacinar, trata do processo social que envolve as conexões entre as pessoas, destacando-se o papel de familiares e líderes comunitários ou religiosos, estabelecendo-se normas sociais (o que se espera que as pessoas façam) e preferências sociais (vacinar para proteger os outros ou não se vacinar porque os outros já se vacinaram)^10^.

Um terceiro domínio é a mudança direta de comportamentos que parte do princípio que a motivação já está presente e que é possível aumentar a vacinação sem necessariamente mudar o que as pessoas pensam/sentem ou o contexto social em que estão inseridas. Esse domínio pode ser trabalhado a partir da construção de intenções favoráveis à vacinação, como manter a vacinação presente no dia-a-dia das pessoas por meio de lembretes e estímulos midiáticos e reduzir barreiras de acesso, bem como a partir de incentivos ou exigências que promovam a vacinação^10^.

Observou-se que intervenções que tentam modificar o domínio do pensar/sentir, embora correlacionado com o recebimento da vacina, apresentam efeitos menos consistentes. Intervenções no domínio do processo social são promissoras, mas ainda requerem maiores evidências. Intervenções no domínio da mudança direta do comportamento apresentam efeitos mais expressivos no aumento da vacinação. Por fim, destaca-se a recomendação pelos profissionais da saúde como a intervenção mais potente^10^.

Em 2022, a OMS publicou um *position paper* sobre os resultados do grupo de trabalho, apresentando uma adaptação do Modelo de Aumento da Vacinação (Figura). A hesitação vacinal foi definida como “um estado motivacional de estar em conflito sobre a, ou de se opor à vacinação; isso inclui intenções e disposição”. Separa a motivação/hesitação do comportamento resultante (recebimento da vacina), de forma que devem ser medidos e compreendidos independentemente. Essa definição substitui a anterior de 2014^1,9^.

**Figura:** Quadro da Organização Mundial da Saúde sobre os determinantes comportamentais e sociais da vacinação, 2022.

Fonte: Adaptado de WHO^9^ (2022).

### Diferentes Fases da Hesitação Vacinal Infantil no Brasil

No Brasil, antes da definição da hesitação vacinal pela OMS, em 2014, e da queda das coberturas vacinais, em 2016, ou seja, momento de elevada cobertura vacinal, a produção científica sobre recusa ou atraso vacinal era mais expressiva em estudos qualitativos de famílias de média e alta renda e evidenciava a determinação do processo social sobre a decisão da (não) vacinação^11,12^.

Com a necessidade de compreender a queda das coberturas vacinais, a partir de 2016, estudos sobre a hesitação vacinal no Brasil cresceram em número e passaram a considerar os modelos de três C e a matriz de determinantes da OMS^13,14^. O estudo de Brown et al.^13^ mostrou que as razões mais comuns para a hesitação foram questões relacionadas à confiança na vacina. Silveira et al.^15^ demonstraram a emergência da hesitação vacinal nas coortes de nascimento de Pelotas (RS), a partir da inversão do padrão do gradiente social de menor cobertura vacinal entre as famílias mais pobres em 1982 para as mais ricas em 2015. Em Campo Grande (MT), Nascimento et al.^14^ encontraram 39% de pais hesitantes, principalmente por falta de confiança na vacina. Garcia et al.^16^ utilizaram o instrumento em escala Likert “*10-item Vaccine Hesitancy Scale*” da OMS^1^, em Araraquara (SP). Distinguiram-se em dois componentes – a confiança na vacina e a percepção de risco a eventos adversos – e destacaram o papel dos profissionais de saúde no enfrentamento da hesitação vacinal, orientando a vacinação por meio de uma relação de confiança^16^.

O Inquérito de Coberturas Vacinais 2020 mostrou que barreiras de acesso e dificuldades programáticas suplantaram a hesitação vacinal como motivo para menor adesão à vacinação. Os dois motivos de acesso mais evidentes e que explicaram a redução de 62% da cobertura vacinal completa foram a falta de dinheiro para transporte e a perda da caderneta de vacinação^6,17^.

Destacam-se também estudos qualitativos, como de Matos et al.^18^, que evidenciou o papel do contexto social, dos profissionais da saúde e acesso à informação como sendo fundamentais para o processo de tomada de decisão sobre a vacinação infantil. A percepção do risco e a confiança na vacina (composição, efetividade e eventos adversos), no PNI (calendário de vacinação), e na indústria farmacêutica, além de estilos de vida e de mundo das famílias, foram elementos-chave para a hesitação vacinal. Cunegundes et al.^19^trabalharam com pais altamente escolarizados de São Paulo (SP) que descartaram o rótulo antivacina. Discutiu-se a (não) vacinação como parte de um conjunto de escolhas que caracterizam uma identidade social mais ampla e quatro temas emergiram das narrativas: estilo de vida natural; pressão social no contexto da vacinação; pandemia e vacinação contra covid-19; e desconfiança nas vacinas, indústria farmacêutica, profissionais de saúde e instituições.

Diversos estudos sobre hesitação da vacina covid-19 apontaram prevalências de hesitação em torno de 20% na população geral^20,21^ e de para grupos específicos como crianças^22^, gestantes^23^, idosos^24^ e condições clínicas específicas^25^. Nesses estudos, para análise de associação, além de variáveis relacionadas a confiança na vacina e percepção de risco da doença, socioeconômicas e demográficas, foram analisadas de forma mais contundente as características das vacinas (tipo de plataforma, laboratórios e país de origem), comorbidades, religião, orientação política e formas de busca de informação, com ênfase na mídia *online* ^20,21,23,26^.

A recomendação da vacinação pelos profissionais da saúde é amplamente reconhecida como uma das ações mais efetivas para o aumento da adesão à vacina. No entanto, a hesitação vacinal entre os profissionais da saúde também já foi alvo de diversos estudos e revela um cenário preocupante, tanto para a própria vacinação^27,28^, quanto para a recomendação da vacinação para a população^29,30^.

Destacam-se, após a pandemia, estudos de hesitação vacinal e orientação política e religiosa^26,31,32^. Estudo de Pereira et al.^26^destacou que a religião exerce papel importante no efeito sobre as preferências políticas e percepção de segurança da vacina. Matos et al.^33^ apresentaram o termo “politização da vacina”, definindo-o como “a influência de interesses e ideologias políticos sobre avaliações técnicas ou científicas das políticas de imunização, com o objetivo de formar ou modificar as opiniões públicas em prol de um interesse político”. Sob a perspectiva de cuidadores de crianças, a politização da vacina ficou evidente com a pandemia de covid-19 e deve ser tratada como um desafio contemporâneo ao contexto brasileiro e considerada na formulação de políticas de imunizações^33^.

A pandemia de covid-19 afetou a credibilidade das instituições científicas e da mídia tradicional, propiciando o avanço de crenças conspiratórias e narrativas autoritárias. Assim, o negacionismo científico comprometeu a adesão à vacinação^33^.

A desinformação sobre as vacinas, diante de uma população hiperconectada, se potencializou com a pandemia. Em inglês, a literatura diferencia os conceitos de “*misinformation*”, como informação equivocada, mas sem intencionalidade, de “*disinformation*”, como informação incorreta com intenção deliberada de enganar/causar danos^34^. Galhardi et al.^34^ encontraram que as plataformas Instagram, Facebook, Twitter e WhatsApp foram os principais meios de compartilhamento de desinformação sobre a covid-19. Da mesma forma, detectaram grande circulação de desinformação sobre vacinas relacionada a polarização política. Estudo sobre motivos de recusa da vacina de covid-19, a partir de análise de postagens no Twitter identificou cinco categorias temáticas: individualidade, receio de eventos adversos, ideologias políticas/aversão às recomendações do Estado, ceticismo quanto à eficácia da vacina e recusa de produtos ou intervenções não naturais. Sugere-se que as redes sociais podem perpetuar atitudes hesitantes e intensificar o isolamento ideológico^35^. Assim, deve-se analisar a desinformação sobre as vacinas também a partir de uma ótica politizada e do negacionismo científico. Ademais, a economia política da desinformação depende de uma infraestrutura de monetização, por meio de estratégias híbridas de captação de recursos (doações, venda de produtos, publicidade e mensalidades/taxa de adesão), para se sustentar financeiramente e obter incentivos econômicos e políticos, o que contribui para a persistência das narrativas antivacinas^36^.

### Novos Desafios e Potencialidades com a Introdução da Inteligência Artificial na Busca de Informação sobre Vacinas

Conforme mencionado ao longo do texto, durante a última década e, principalmente, após a pandemia de covid-19, as características da hesitação vacinal no Brasil mudaram de forma substancial, influenciadas por diversos fatores, sendo um deles a forma de como as pessoas buscam e têm acesso às informações.

A inteligência artificial permite identificar a desinformação e discernir padrões e sentimentos que influenciam a adesão à vacina. Pode ser utilizada para a construção de estratégias de comunicação inovadoras de combate à desinformação sobre vacinas, considerando grupos mais vulneráveis, com dificuldade de acesso digital, e adaptações segundo traços psicológicos^37^.

As redes sociais tornaram-se importantes fontes difusoras de informações e desinformações sobre vacinas. Estudo de análise de sentimentos, a partir de um modelo de linguagem natural, mostrou capacidade preditiva de tweets em larga escala e em tempo real nas coberturas da vacina covid-19, demonstrando o potencial da ferramenta para a elaboração de estratégias de identificação de focos de hesitação vacinal^38^.

Salas et al.^39^ analisaram as respostas do ChatGPT sobre 50 mensagens falsas mais prevalentes, contraindicações e mitos sobre vacinas que circulavam na internet em 2023 e concluíram que, embora a ferramenta não possa substituir um especialista, ela possui potencial a ser explorado na orientação à vacinação, se utilizar dados alinhados às evidências científicas.

Ensaios randomizados controlados apontaram estratégias promissoras de *chatbots* para diminuir a hesitação vacinal, aumentar a confiança na vacina e corrigir informações equivocadas sobre a vacinação em diversas regiões no mundo^40^. Brown et al.^40^ mostraram que um *chatbot* no Whatsapp com mensagens interativas bidirecionais, com funcionalidades comportamentais, aumentou em mais de três vezes a vacinação contra covid-19 em relação ao grupo sem intervenção e quase dobrou a vacinação se comparado com a estratégia de lembrete de vacinação unidirecional. Estudo de Hou et al.^41^ mostrou a efetividade de um *chatbot* voltado para vacinação contra o papilomavírus humano entre meninas do ensino fundamental na China. O *chatbot* foi construído por tecnologias linguísticas avançadas por meio do GPT-4 e baseou-se em informações de órgãos de autoridades de saúde. Os resultados indicaram que o *chatbot* aumentou a vacinação das estudantes e melhorou o letramento sobre vacinas entre os pais. Baudoin et al.^42^também estudaram o efeito da intervenção por *chatbot* em estudantes adolescentes, na França. Observaram melhora das atitudes sobre a vacinação e diminuição de crenças equivocadas. Resultado importante também foi que esse efeito não dependeu da confiança dos participantes na ciência, de suas atitudes iniciais em relação à vacinação ou de sua capacidade acadêmica. Lu et al.^43^ demonstraram o potencial da geração de mensagens assistida por inteligência artificial para elaborar mensagens corretivas eficazes, especialmente quando direcionadas a traços de personalidade como a extroversão. No entanto, as mensagens que abordam crenças profundamente enraizadas foram ineficazes ou apresentaram efeito reverso, ressaltando a necessidade de supervisão humana no refinamento dessas mensagens.

Em outubro de 2025, a Organização Pan-Americana da Saúde^44^ lançou um guia com recomendações de *prompts* para inteligência artificial generativa que se possa produzir conteúdo em saúde pública confiável e adequado a diferentes culturas e contextos, de forma a contribuir a comunicação e tomada de decisão em saúde pública. No Brasil, como experiência concreta, o Ministério da Saúde lançou o assistente virtual no WhatsApp em dezembro de 2023, como parte do programa interministerial Saúde com Ciência, que tem como foco a valorização da ciência e disseminação de informações confiáveis. O *chatbot* tem como objetivo facilitar o acesso a informações corretas sobre a vacinação (campanhas e calendários) e vacinas (características, benefícios e importância) e o enfrentamento da desinformação.

Ademais, faz-se necessário explicitar desafios para o uso dessa ferramenta num contexto de iniquidade de acesso a tecnologias, conectividade e letramento digital. No Brasil, estudo apontou importante heterogeneidade regional, demográfica e social de acesso à internet, com maior exclusão digital entre pessoas idosas, populações em situação de pobreza e residentes de áreas rurais, sendo que os motivos para menor uso incluíram falta de interesse, conhecimento, disponibilidade e custo^45^. Como consequência, destaca-se o viés do algoritmo ao utilizar dados que não são representativos da população-alvo para treinar e testar os sistemas de inteligência artificial, perpetuando discriminação e exclusão sistemáticas. Da mesma forma, devem ser mencionados outros vieses relacionados a opacidade, privacidade e governança dos dados. Por fim, cabe ressaltar o impacto ambiental e social associado ao ciclo de vida dos sistemas de inteligência artificial, incluindo recursos energéticos e mudanças da organização de trabalho^46^.

Assim, com o uso cada vez mais comum e de fácil acesso à inteligência artificial, instala-se um novo desafio ao manejo da adesão à vacinação. Por um lado, é possível utilizar essa ferramenta para o aumento do acesso a informações corretas sobre vacinas e assim reduzir a hesitação vacinal, por outro, pode gerar mais desinformação e iniquidades à vacinação ao não considerar as limitações de dados e algoritmos utilizados^37^.

## CONSIDERAÇÕES FINAIS

A hesitação vacinal é um fenômeno dinâmico e movido pelas mudanças do contexto social e epidemiológico. Neste comentário, registrou-se a evolução do conceito, bem como a forma que ele foi abordado na literatura científica brasileira após o início das quedas de cobertura vacinal em 2016 e após o início da pandemia de covid-19.

A inteligência artificial terá grande impacto no desenvolvimento de estratégias para aumento da vacinação e deve-se considerar um contexto de iniquidade social e os desafios de governança e externalidades da ferramenta. Destaca-se, portanto, a necessidade de desenvolver ferramentas de inteligência artificial para a identificação, monitoramento e esclarecimentos sobre conteúdos relacionados à vacinação, em conjunto com formas educativas ao público usuário para adequada utilização, fortalecendo a confiança nas vacinas e a redução da hesitação vacinal.

## Data Availability

não se aplica.
